# Postoperative brachytherapy alone for 217 patients with early-stage oral cavity squamous cell carcinoma

**DOI:** 10.1016/j.ctro.2025.100922

**Published:** 2025-01-16

**Authors:** C. Schweizer, V. Strnad, M. Lotter, S. Kreppner, R. Merten, R. Fietkau, A. Karius

**Affiliations:** a Department of Radiation Oncology, Universitätsklinikum Erlangen, Friedrich-Alexander-Universität Erlangen-Nürnberg (FAU), Erlangen, Germany; b Comprehensive Cancer Center Erlangen-EMN (CCC ER-EMN), Erlangen, Germany

**Keywords:** Early-stage oral cavity cancer, Postoperative radiotherapy, Interventional radiotherapy, Long-term toxicity after brachytherapy, HDR, PDR

## Abstract

**Introduction:**

Postoperative radiotherapy is generally recommended for pT1/2pN0 squamous cell carcinoma of the oral cavity (OSCC) if risk factors are present. Prospective studies are missing. Interventional radiotherapy offers a precise dose application. In this paper we analyze long-term efficacy and toxicity in a large single-center cohort of patients with early OSCC receiving sole postoperative brachytherapy (BT).

**Material and methods:**

From 1998 to 2023, 217 patients were postoperatively treated with sole BT in our institute. The median follow-up was 110 months (range: 2–316). The primary objective was local control. Secondary outcomes were overall survival, cancer specific survival, and toxicity.

**Results:**

The local recurrence rates for 12, 24, and 60 months were 7.1 %, 9.1 %, and 12.6 %. The disease-free survival was 89.7 %, 86.1 %, and 79.3 %. The overall survival rates at 12, 24, and 60 months were 94.4 %, 89.6 %, and 77.9 %. The cancer-specific survival was 97.1 %, 96.6 %, and 92.9 %, respectively. At two years, the rate of regional recurrence was 8.3 %. Patients without neck dissection had a significantly increased risk for lymph node recurrence (p = 0.025). Side effects ≥ grade 3 were seen in 14 % (30/217). 17 % (37/217) of patients developed a soft tissue necrosis (STN). Osteoradionecrosis (ORN) was seen in 7 % (15/217) of patients. A target volume > 15 cm^3^ was significantly associated with the occurrence of STN (p = 0.011) and ORN (p = 0.004).

**Conclusions:**

Postoperative interventional radiotherapy for previously not irradiated patients with early-stage OSCC is a safe and efficient treatment. Randomized trials are needed to compare these results to omission of postoperative radiotherapy as well as external beam radiotherapy.

## Highlights

• Postoperative interventional radiotherapy for early squamous cell cancer of the oral cavity (OSCC) is safe and efficient.
• After neck dissection, limitation of dose application to the tumor bed is not associated with increased regional failure.
• Compared to external beam radiotherapy, brachytherapy renders possible precise dose application with less toxicity.
• There is still a substantial lack of prospective data and precise guidelines for the postoperative treatment of early OSCC.

## Introduction

For patients diagnosed with squamous cell cancer of the oral cavity (OSCC), surgery is generally the primary treatment. Radiotherapy and systemic therapies like chemotherapy and immunotherapy are established treatment options depending on the tumor stage and histopathological findings [1], [2], [3]. Brachytherapy (BT) for head and neck malignancies is a well-established treatment [4]. For early-stage OSCC (T1-2 N0), the optimal treatment has not yet been clarified. In certain circumstances, BT alone is considered a valid treatment [5]. After complete surgical resection of the primary tumor, treatment of the neck and the indication for local adjuvant therapy remain to be decided. An adequate ipsilateral neck dissection and in case of the primary tumor crossing the midline also a contralateral neck dissection is strongly recommended. There is no broad consensus on which risk factors are most relevant for the oncologic outcome. Certain risk factors are well known, like close resection margins, poor differentiation, lymphovascular invasion, and the depth of tumor invasion [6], [7], [8]. Several retrospective reports evaluating postoperative local treatment after resection in pN0 diseases show excellent local control with acceptable rates of treatment toxicity [9], [10], [11], [12]. However, current guidelines and expert consensus statements lack clear recommendations [6], [13], [14], [15].

For postoperative radiotherapy, different treatment options are available. External beam radiation therapy (EBRT) in the head and neck region is accompanied by relevant radiation dose in the surrounding healthy tissue with the risk of severe side effects. The impact on quality of life when harming the swallowing apparatus, for example, gained more interest over the last few years [16]. Therefore, it’s important to explore the possibility of reducing the irradiated volume. Multicatheter BT can apply a high dose to the tumor bed meanwhile sparing the surrounding organs at risk due to the steep dose gradient [17]. In literature, several BT treatments are reported utilizing the low-dose rate (LDR), high-dose rate (HDR), and also pulsed-dose rate (PDR) regime. Perioperative BT is performed in several centres as well [18]. The identification of those patients who will benefit from the postoperative radiation of the tumor bed is crucial. Of course, patients with an indication for chemotherapy or irradiation of the lymphatic drainage do not qualify for sole BT of the tumor bed. However, with the presence of relevant risk factors like depth of infiltration or poor tumor differentiation, interstitial BT can offer an effective treatment and the simultaneous protection of the surrounding organs at risk [17].

In the past, we already published several analyses including data of all of our patients who received BT of the head and neck [19], [20], [21]. This specific subgroup of early OSCC in the postoperative setting has not yet been studied in detail. In the present analysis, we describe our single-institutional results of postoperative interstitial BT for squamous cell cancer of the tongue and of the floor of the mouth. Tumor control, survival outcome, toxicity and treatment details for PDR- and HDR-brachytherapy using Iridium-192 were evaluated.

## Material and methods

### Study population

We retrospectively evaluated all patients treated in our institution with sole postoperative interstitial BT for early-stage OSCC in curative intent from 1998 to 2023. Due to the retrospective character of the study and the analysis of only in-house data, no ethics approval had to be obtained according to our local ethics committee. The inclusion criteria were sole postoperative BT of the tongue or of the floor of the mouth without EBRT and without systemic therapy. No prior radiation of the head and neck region was allowed. In total, 217 patients met the inclusion criteria. Patient and tumor characteristics are listed in Table 1. The median age was 56 years (range: 28–83), 71 % (155/217) of patients were male. The tumor localization was the mobile tongue in 53 % (115/217) of the patients and the floor of the mouth in 47 % (102/217).

**Table 1:** Patient characteristics (n = 217).

Characteristic	No. of patients	%
Age (y)	Median: 56, range: 28–83	
<60	130	60
≥60	87	40
Sex		
Female	62	29
Male	155	71
Tumor site		
Mobile tongue	115	53
Floor of the mouth	102	47
Tumor grading (G)		
G1	20	9
G2	136	63
G3	59	27
unknown	2	1
pT classification (tumor size, AJCC 8)		
pT1	72	33
pT2	107	49
pT3	34	16
pT4	4	2
Lymphangiosis (L)		
L0	146	67
L1	52	24
unknown	19	9
Vascular invasion (V)		
V0	151	70
V1	3	1
Unknown	63	29
Perineural invasion (Pn)		
Pn0	47	22
Pn1	15	7
Unknown	155	71
Resection margin (R)		
R0	198	91
>5 mm	24	11
1 mm–5 mm	21	10
<1 mm	4	2
No further details except R0	149	69
R1	7	3
unknown	12	6
Depth of infiltration (mm)	Median: 7, range: 1–22	
≤5	59	27
6–10	71	33
>10	35	16
unknown	52	24
Neck dissection		
Ipsilateral +/- contralateral	202	93
No neck dissection	15	7

The tumor classification changed in 2017, when the depth of tumor invasion was included into the T stadium. Therefore, 185 patients were additionally re-staged according to the 8th AJCC classification. An up-staging was seen in 41 % of patients (76/185). The most common tumor grade was G2 (63 %, 136/217), followed by G3 (26 %, 57/217). According to the 8th AJCC classification, 49 % (107/217) of the patients were classified as T2. Four patients (2 %) were classified as T4 due to erosion of the periosteum. The depth of invasion was available for 77 % (167/217) of patients and showed a median of seven millimeters with a range from one to 22 mm. A neck dissection was performed in 92 % (200/217) of patients. The state of lymphatic invasion was documented for 91 % (198/217) of patients. Lymphangiosis was present in 24 % (52/217) of patients.

### Treatment

After tumor resection, certain histopathological results lead to the indication for postoperative brachytherapy. These included close resection margins, poor differentiation, lymphangiosis, vascular invasion, and the depth of tumor invasion. A team of head and neck specialists or maxillofacial surgeons, pathologists, and radiation oncologists decided on the treatment recommendation in a tumor board conference. The median time interval from tumor resection to brachytherapy was 68 days (range: 12–177).

**Fig. 1:**
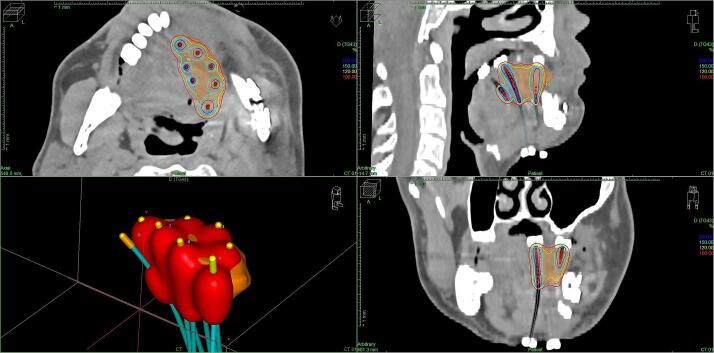
Clinical example of a 54-year-old patient with cancer of the oral cavity treated with postoperative brachytherapy after tumor resection and local flap surgery due to a depth of tumor infiltration of 8 mm. Axial, sagittal and coronal view of the planning ct scan after implantation of eight single-leader brachytherapy catheters. Red: 100 % isodose, yellow: 120 % isodose, turquoise: 150 % isodose, blue: 200 % isodose. Lower image to the left: 3-D aspect of the 100 %-isodose (red) and the brachytherapy catheters (turquoise). (For interpretation of the references to colour in this figure legend, the reader is referred to the web version of this article.)

The insertion of the brachytherapy catheters was performed under general anesthesia. A median of nine catheters (range: 5–16) was implanted. The target volume was determined combining the information of initial imaging, reports of prior surgeries, clinical examination, and a computed tomography (CT) scan. The clinical target volume (CTV) consisted of the palpable and visible tumor bed with an additional margin of 5–10 mm in all directions meanwhile respecting natural borders like bone or skin. No additional margin was added for the planning target volume (PTV; CTV = PTV). The median CTV was 14.6 cm^3^ (range: 5.1–43.2). The treatment was delivered in the HDR- or the PDR-regime using Iridium-192. The majority of patients received PDR-BT (91 %, 198/217). Fig. 1 provides a clinical example for the BT dose distribution. All treatment schedules are displayed in Table 2. The median physical dose for PDR-BT was 56.65 Gy (range: 36.5–64 Gy) in 103 (range: 64–120 Gy) fractions of 0.55 Gy (range: 0.45–0.7 Gy). The median physical HDR-dose was 34.2 Gy (range: 30–40.8 Gy) in 10 (range: 8–12) fractions of 3.4 Gy (range: 3–4 Gy). Assuming a value of alpha/beta = 3 Gy and calculating the biological effective doses equivalent to the application of single fractions of 2 Gy (EQD2), this would correspond to a calculated median total equivalent dose of 43.8 Gy for the HDR treatment. For HDR-BT, the median physical D_90_ was 3.8 Gy (range: 3.4–4.3 Gy) and the median V_100_ was 95.1 % (range: 90.0–97.2 %). For PDR, the median D_90_ was 0.59 Gy (range: 0.48–0.71 Gy) and the median V_100_ was 93.3 % (range: 65.0–99.3 %).

**Table 2:** Treatment details for postoperative brachytherapy of the oral cavity.

	HDR: median, range	No. of patients with available data	PDR: median, range	No. of patients with available data
No. of fractions	10, 8–12	19/217, 9 %	103, 64–120	198/217, 91 %
Single physical dose	3.4, 3,0–4	19/217, 9 %	0.55, 0.45–0.7	198/217, 91 %
Total physical dose	34.2, 30–40.8	19/217, 9 %	56.65, 36.5–64	198/217, 91 %
Volume of CTV (cm^3^)	9.8, 5.1–28.2	18/217, 8 %	15.7, 5.2–43.2	102/217, 47 %

Abbreviations: HDR, high dose rate; PDR, pulsed dose rate; CTV, clinical target volume.

Until 2011, treatment planning was performed with the PLATO BPS version 13.1 or 14.0 (Nucletron, Elekta, Netherlands) after catheter reconstruction on planar x-ray images obtained from an integrated brachytherapy unit. In 2007, a planning CT-scan became the new standard. From 2011 on, catheter reconstruction and dose calculation were performed using the Oncentra Brachy planning system, currently version 4.6.3 (Nucletron, Elekta, Netherlands).

### Follow-up

Generally, all patients were scheduled for follow-up visits as follows: six weeks after BT, then every three months for the first two years after BT, then every six months for the 4th and 5th year. After five years, the follow-up was continued once yearly. Patients were routinely seen by a radiation oncologist as well as an otolaryngologist or a maxillofacial surgeon. Follow-up visits included anamnesis, clinical examination, and computed tomography scans. Side effects were judged according to the toxicity criteria of the Radiation Therapy Oncology Group (RTOG) and the European organization for research and treatment of cancer (EORTC) [22].

### Statistical analysis

Recurrence and survival rates were calculated using the Kaplan-Meier-method. Toxicity rates were evaluated using the Chi-squared test and the Fisher’s exact test. Differences in-between groups were determined by the log-rank test. Possible prognostic factors for local recurrence were additionally calculated using the Cox regression model. The statistical analysis was performed with IBM SPSS Statistics (version 28.0.0.0; IBM, USA).

## Results

### Tumor control and survival outcome

**Fig. 2:**
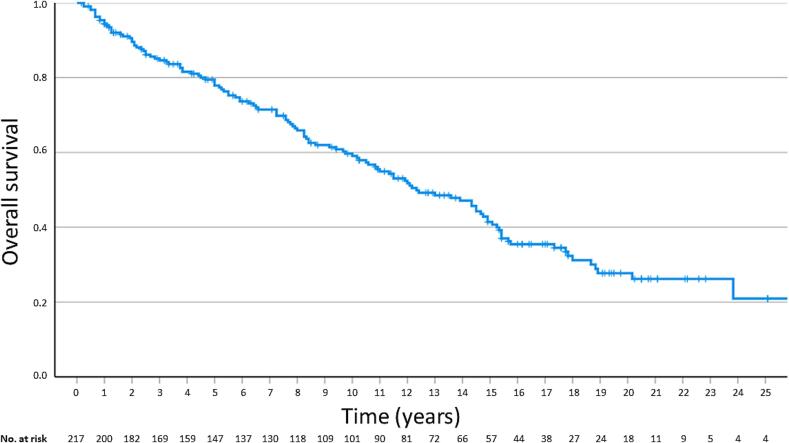
Local recurrence rate for patients with early-stage squamous-cell cancer of the oral cavity treated with postoperative brachytherapy.

**Fig. 3:**
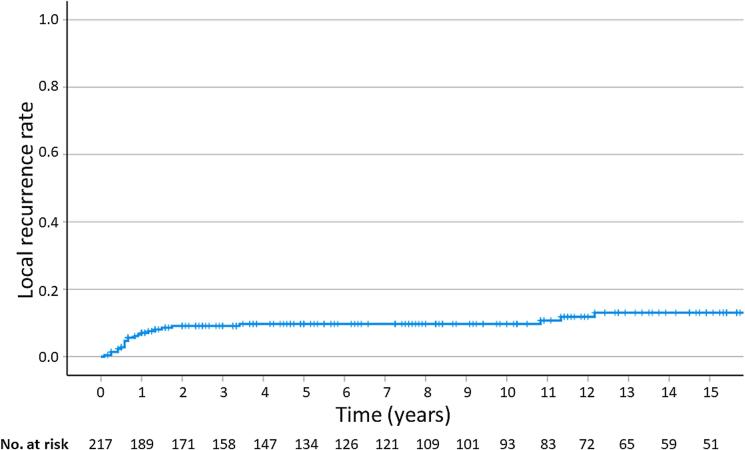
Overall survival for patients with early-stage squamous-cell cancer of the oral cavity treated with postoperative brachytherapy.

The median follow-up was 110 months, with a range of two to 316 months. The LRR for 12, 24, 60, and 180 months were 7.1 %, 9.1 %, 9.7 %, and 13.1 % (Fig. 2). The disease-free survival (DFS) was 89.2 %, 85.7 %, 81.2 %, and 75.9 %, respectively. The overall survival (OS) at 12, 24, 60, and 180 months was 94.4 %, 89.6 %, 77.9 %, and 41.4 % (Fig. 3). The cancer-specific survival (CSS) was 97.6 %, 97.1 %, 93.6 %, and 89.6 %, respectively. The estimated rates at two years for regional recurrence and distant metastasis were 8.3 % and 5.4 %, respectively. At five years, these rates were 10.5 % and 7.2 %. The cumulative rates of a second malignancy at five and 15 years were 7.6 % and 21.9 %.

Regarding the LRR at two years, pT3-4 patients had a significantly worse outcome than pT1-2 patients (18.6 % vs. 7.1 %, p = 0.026, hazard ratio [HR] 1.699 [95 % CI 1.066 to 2.708], Table 3). A tumor grading of G1 or G2 vs. G3 was also highly significant for the LRR at two years: 5.9 % vs. 18.1 % (p = 0.013, HR 3.143 [95 % CI 1.277 to 7.740]). Patients with a depth of tumor invasion of more than 10 mm had a significantly worse LRR at two years (p = 0.046, HR 2.945 [95 % CI 1.022 to 8.490]). As displayed in Table 3, other factors showed no statistical significance for the LRR at two years. In the multivariate analysis for the statistically significant factors, only pT1-2 vs. pT3-4 remained a statistically significant factor (p = 0.016).

**Table 3:** Local recurrence rate at two years in dependance of risk factors.

Risk factor	No. of patients	Two-year LRR	p-value	Hazard ratio (0.95 % confidence interval)
T1/2 vs. T3/4	179 vs. 38	7.1 % vs. 18.6 %	**0.026**	1.699 (1.066 to 2.708)
G1/2 vs. G3	156 vs. 59	5.9 % vs. 18.1 %	**0.013**	3.143 (1.277 to 7.740)
Depth of infiltration ≤10 mm vs. >10 mm	130 vs. 35	6.5 % vs. 17.2 %	**0.046**	2.945 (1.022 to 8.490)
Lymphangiosis L0 vs. L1	146 vs. 52	10.8 % vs. 5.8 %	0.390	0.580 (0.168 to 2.005)
Vascular invasion V0 vs. V1	151 vs. 3	9.1 % vs. 0 %	0.742	0.048 (0.000 to 3267861.920)
Perineural invasion Pn0 vs. Pn1	47 vs. 15	18.7 % vs. 15.2 %	0.775	0797 (0.169 to 3.756)
Positive resection margin R0 vs. R1	198 vs. 7	9.5 vs. 16.7 %	0.650	0.627 (0.084 to 4.698)

P-values < 0.05 were bolded.

We also compared the patients with a documented R1-resection (7/205, 3.4 %) to those with a documented R0-resection (198/205, 97 %). No statistical significance for the 2-year-LRR (p = 0.650), DFS (p = 0.673), and CSS (p = 0.551) was found. Of 217 patients, 15 patients (6.9 %) had no neck dissection. There were no significant differences for the LRR (p = 0.639), DFS (p = 0.554) or CSS (p = 0.857) for those 15 patients. Looking specifically at the risk of developing lymph node metastasis, the patients without initial neck dissection had a significantly increased risk (p = 0.025). Comparing PDR-BT to HDR-BT, no statistically significant differences for LRR, DFS, and CSS and toxicity rates were found. For 32/217 (14.7 %) of the patients, a second malignancy was recorded.

### Side effects

In general, late side effects were limited to xerostomia and dysphagia grade 1–2. Side effects ≥ grade 3 were recorded in 14 % (30/217) of patients. Soft tissue necrosis (STN) was documented in 37/217 (17 %). Surgical treatment for STN was performed in 16/217 (7 %) of patients (STN grade 3). 21/217 (10 %) of patients with a STN grade 2 were treated conservatively. The median time from BT to the occurrence of STN was 8 months (range: 1–188 months). The rate of osteoradionecrosis (ORN) was 7 % (15/217), all of them requiring surgical treatment (ORN grade 3). The median time from BT to the occurrence of ORN was 35 months (range: 4–219 months). No grade 4 or 5 toxicity was recorded. The size of the CTV was documented only for the time after the implementation of planning CT-scans. Therefore, CTV data was available for 55 % (120/217). The median CTV volume was 14.7 cm^3^ (range: 5.1–43.2 cm^3^). A volume larger than 15 cm^3^ was significantly associated with the development of a STN (p = 0.011) as well as with the occurrence of ORN (p = 0.004). No toxicity regarding the skin was observed.

## Discussion

The available literature on postoperative radiotherapy for OSCC typically includes a very heterogenous patient selection, which is probably due to low patient numbers in most departments. Just recently, Renard et al. published their results for 66 patients treated with BT [12]. Comparability to our findings is limited since their patient collective is mixed: some patients received additional EBRT. With a local control (LC) of 87 % at five years, our result with a five-year local recurrence rate of 9.7 % is even better. Already in 2004, Lapeyre et al presented data of pT1-2 N0 patients treated with postoperative BT [23]. Similar to our results at five years, OS, CSS, and LC in this subgroup were 75 %, 85 %, and 88 %, respectively. Only very limited data is published describing the outcome for patients without any adjuvant treatment despite risk factors present. Shim et al. reported a local control of 100 % five years after postoperative brachytherapy, compared to 82 % without adjuvant treatment in a retrospective analysis [24]. Other reports could not quite confirm these excellent results, with local control rates after adjuvant brachytherapy of 85 % to 88.5 % [9], [11]. Again, these cohorts were quite heterogeneous and not balanced due to the retrospective character of the analyses.

For external beam radiotherapy, some data is available. In their retrospective analysis, Ganly et al. reported neck recurrences for about 18 % in patients with OSCC T1-2 N0 treated without postoperative radiation [25]. Importantly, they did not look at the DOI. Patients with a pT3 stadium were also included and received no further treatment. In our cohort, the regional recurrence-free survival at five years was 89.5 %, compared to 79.9 % reported by Ganly et al. Another large retrospective cohort was published just recently by Tian et al [26]. In 528 patients diagnosed with pT1-2 N0 oral tongue SCC, 27.5 % received postoperative radiation of the tumor bed and of the bilateral lymphatic drainage. It’s worth mentioning that almost a quarter of these patients had no neck dissection. A propensity score matching of the patients with surgery and radiation vs. the patients with surgery only was performed. The LRR at five years was 12 % vs. 26 % and the regional recurrences were 16 % vs. 28 %. Our results at five years with a LRR of 9.7 % and a regional recurrence rate of 10.5 % are both better. The authors conclude postoperative radiation is beneficial for patients with G2 and G3 differentiation, perineural or lymphovascular invasion as well as a DOI of more than 5 mm. There is no data regarding treatment toxicity. It remains unclear, if all these patients actually require radiation of the bilateral lymphatic drainage, especially if adequate neck dissection was performed. At the same time, the long-term toxicity of EBRT in the head and neck area is highly relevant and should always be considered when recommending EBRT. Interestingly, both studies report worse regional control at five years after EBRT including the lymphatic drainage of 20.1 % and 16 % vs. only 10.5 % in our cohort.

Concerning long-term toxicity, STN and ORN are considered most relevant. After EBRT, ORN is a well-known issue as well [27]. In a dosimetric comparison of EBRT and BT, clearly the least dose was distributed in the mandible when using BT [17]. Our rates of STN (17 % in total, surgical treatment for 7 %) and ORN (7 %) are quite in line, even a bit lower than results of previous studies [18], [20], [28], [29]. Other studies reported lower toxicity rates of 8–9 % for STN and 2–2.4 % for ORN [12], [18], [30]. Importantly though, treatment setting and brachytherapy techniques vary within the published cohorts. Even when following specific dose constraints, a certain rate of STN and ORN seems to be unavoidable. In our cohort, occurrence of both STN and ORN were significantly increased with a CTV > 15 cm^3^. The median CTV in our cohort was 14.7 cm^3^, which is comparable to other published data [17]. Patients must be informed of this risk before treatment.

Importantly, the inclusion of the depth of tumor invasion (DOI) in the T-state affected many patients. In our cohort, 41 % of patients were re-classified as a higher T-stadium due to the DOI. On the other hand, our data shows significantly higher rates of local recurrence within two years for T3-4 compared to T1-2. These results remained statistically significant also in the multivariate analysis. Therefore, our results confirm the relevance of the T-stages for tumor control. Patients classified with a pT3-4 tumor stage should be treated not only with postoperative brachytherapy.

The limitations of this study are mainly due to its retrospective character. Not all data was available, e.g., pathological findings were not complete. Some patients who were treated with sole postoperative interstitial brachytherapy were in fact not perfectly suitable. Only 92 % had a neck dissection. Since the risk of microscopic lymph node involvement is quite high for OSCC, a neck dissection is considered mandatory [31]. OSCC is generally known for a higher risk of lymphatic spread than oropharyngeal cancer, as the results of the just recently published prospective DIREKHT trial confirm [32]. Four patients with a T4-stadium were treated with sole brachytherapy, which is not in accordance with current guidelines. This might have had a negative impact on the survival results. There was no randomization, therefore, a potential bias in the choice of treatment is possible. Not all patients were treated with the same technique. PDR vs. HDR regime and the switch to CT-based planning in 2007 might have affected the results, which has to be investigated in further studies. Since we only started in 2019 using HDR-BT for head and neck patients more frequently, a comparison of PDR-BT and HDR-BT is difficult. For detailed analysis of BT treatment toxicity, thorough documentation of the status after the surgical treatment and before BT as a baseline would be necessary. Taken together, a prospective study setting is needed.

Nevertheless, we present a large patient cohort for this specific indication treated with BT, to our knowledge even the largest one published so far. Seeing the substantial lack of prospective data, randomized studies need to clarify which patients really benefit from sole postoperative brachytherapy and which patients don’t need adjuvant treatment after surgery.

## Conclusions

Interventional radiation therapy after surgery for patients with early-stage squamous cell cancer of the tongue and the floor of the mouth with relevant risk factors showed to be a safe and efficient treatment. The risk of developing STN or ORN was increased with a CTV > 15 cm^3^. Randomized trials are required to allow comparing these results to the outcome after surgery alone and to postoperative EBRT.

## CRediT authorship contribution statement

**C. Schweizer:** Conceptualization, Formal analysis, Methodology, Project administration, Writing – original draft. **V. Strnad:** Conceptualization, Resources, Supervision, Writing – original draft. **M. Lotter:** Writing – original draft. **S. Kreppner:** Writing – original draft. **R. Merten:** Writing – original draft. **R. Fietkau:** Resources, Supervision, Writing – original draft. **A. Karius:** Writing – original draft.

## Funding

There was no external funding.

## Declaration of competing interest

The authors declare that they have no known competing financial interests or personal relationships that could have appeared to influence the work reported in this paper.
